# Supplementary material for: Selecting the Appropriate Downstaging and Bridging Therapies for Hepatocellular Carcinoma: What Is the Role of Transarterial Radioembolization? A Pooled Analysis
Source: Cancers (Basel). 2023 Apr 2;15(7):2122. doi: 10.3390/cancers15072122 (PMC10093460; doi:10.3390/cancers15072122)
Supplement: Supplementary file 1 [file cancers-15-02122-s001.zip › cancers-2247512-supplementary.pdf]

Supplementary Table S1 Inclusion criteria for TARE before LT

**Downstaging**	
Iñarrairaegui et al. [13]	Progress on prior TACE; not good candidates for TACE; expected tumor response with segmental radioembolization; Child-Pugh class A; ECOG 0-2; absence of distant metastases; uncorrectable risk of microspheres misplacement into the GI tract
Pracht et al. [14]	Unilobar disease; portal vein thrombosis
Gramenzi et al. [15]	Child–Pugh class A/B; ECOG 0-1; Life-expectancy >3 months; bilirubin ≤2mg/dl; BCLC B/C; granulocyte count ≥$1.5x10^9$/L; platelet count ≥50 $x10^9$/L; tumor extension <50% of liver; no extrahepatic metastasis, previous radio- or chemotherapy, evidence of hp shunt >20% (99mTc-MAA) or evidence of 99mTc-MAA delivery to the stomach or duodenum after embolization of the GDA
Labgaa et al. [16]	Unresectable HCC; multiple nodules confined to the liver; exclusive treatment of TARE without previous treatment other than TARE; MDT
Mehta et al. [17]	HCC exceeding Milan criteria but meeting one of the following (single lesion 5.1-8 cm, 2-3 lesions each ≤5 cm with the sum of the maximal tumor diameters ≤8 cm, 4-5 lesions each ≤3 cm with the sum of the maximal tumor diameters ≤8 cm; no vascular invasion); no extrahepatic disease; bilirubin ≤4 mg/dl; MDT
Serenari et al. [18]	ECOG 0-1; Child-Pugh score ≤B7; portal vein thrombosis limited to the first-order portal branch; no macrovascular invasion or extrahepatic disease
Dhondt et al. [19]	BCLC stage B, extended to patients with BCLC stage A HCC not amenable to ablation, partial hepatectomy, or transplant; less than 50% liver involvement; no extrahepatic disease, invasion of the main, right, or left portal vein; bilirubin ≤34 mmol/L, or ≤44 mmol/L in case of a single involved segment; Child-Pugh score ≤7.
**Bridging**	
Mantry et al. [20]	Unresectable disease; ECOG 0-2; platelets >60,000; creatinine <2 mg/dL; bilirubin <2 mg/dL; INR <1.2; no extrahepatic disease; contraindication to hepatic artery catheterization such as vascular abnormalities; no efractory ascites; uncorrectable flow to the GI tracts; shunt fraction of 20% or greater to the lung; MDT
Radunz et al. [21]	MDT
Zori et al. [22]	MDT
**Mixed**	
Tohme et al. [23]	ECOG 0-1; serum total bilirubin <2.0 mg/dL; adequate renal and hematologic function; no significant pulmonary shunting
Abdelfattah et al. [24]	Surgically unresectable HCC; no extrahepatic disease or macrovascular invasion; Child-Pugh score <10; ECOG 0-2; platelet count >50 x 10^9/L; INR <1.5; creatinine <100 mmol/L; mapping angiography; stimated radiation doses to lungs >20 Gy in a single administration or 30 Gy in multiple administrations
Ettorre et al. [25]	Unresectable disease predominately involving the liver; granulocytes >1,500/mL; platelets >60,000/mL; total bilirubin ≤ 2.0 mg/dL; GOT/GPT/AP less than 5 times the upper limit of normal; forced expiratory volume in 1s >1 L; no pulmonary shunt greater than 20% of ^{99m}Tc-MAA, uncorrectable delivery to the GI tract or complete PVT; MDT
Gabr et al. [26]	MDT

TACE: trasarterial chemoembolization, ECOG: Eastern Cooperative Oncology Group, GI: gastrointestinal, BCLC: Barcelona Clinic Liver Cancer staging system, GDA: gastroduodenal artery, TARE: transarterial radioembolization, MDT: multidisciplinary team, HCC: hepatocellular carcinoma, INR: international normalised ratio; Gy: gray, GOT: glutamic-oxaloacetic transaminase, GPT: glutamate pyruvate transaminase, AP: alkaline phosphatase.

Supplementary Table S2. Comparison with other therapies

TARE: transarterial radioembolization, TACE: chemoembolization, DEB: drug-eluting bead, LT: liver transplantation, AE: adverse event, BCLC: Barcelona Clinic Liver Cancer staging system, HCC: hepatocellular carcinoma, AFP: alpha-fetoprotein, OS: overall survival, FS: free survival

	**Comparative treatments**	**Effectiveness on HCC**	**Adverse events**	**Outcomes**
**Downstaging**				
Gramenzi et al. [15]	TARE (n=32) vs Sorafenib (n=32)	Downstaging allowing LT only occurred after TARE.	AEs were more frequent with sorafenib therapy: any grade AEs occurred in 91% sorafenib patients and in 59% TARE patients (p<0.0001).	In cirrhotic patients with intermediate-advanced or not otherwise treatable HCC, sorafenib and TARE provide similar survivals.
Mehta et al. [17]	TARE (n=62) vs TACE (n=132)	There were no differences in mRECIST response, probability of or time to successful downstaging, waitlist dropout or LT.	-	There was no significant difference in OS between TARE and TACE.
Dhondt et al. [19]	TARE (n=32) vs DEB-TACE (n=34)	-	In the TARE arm, 39% experienced at least one serious AEs ≥ grade 3 compared with 53% in the DEB-TACE arm (p=0.47).	Resulted in superior tumor control and survival in participants with non-surgical BCLC stage A and B HCC.
**Bridging**				
Zori et al. [22]	TARE (n=28) vs TACE (n=37)	There were no statistical differences in baseline pre-LT characteristics and tumor recurrence. The mVI was seen in 3.6% explants in the TARE group compared with 27% in the TACE group (p=0.013).	-	The TARE group required fewer LRTs (p=0.001) despite no difference in time on the transplant list. There was a trend toward improved 3-year survival in the TARE group (p=0.052).
**Mixed**				
Ettorre et al. [25]	TARE (n=22) vs non-TACE (n=121)	-	-	The OS and FS analysis after LT between TARE and non-TARE were not significant (p=0.113, p=0.897, respectively).
Gabr et al. [26]	TARE (n=93) vs TACE (n=79)	A biological response assessed by AFP decrease was observed in both groups with being more pronounced in the Y90 group.	-	Despite longer time to LT for TARE (p=0.0215), post-LT outcomes were similar between patients with TACE and TARE (p=0.5654).
